# A rare case report of breast sarcoma and synchronous thymoma in a 60-year-old woman

**DOI:** 10.1016/j.radcr.2024.04.077

**Published:** 2024-05-17

**Authors:** Marina Balbino, Federica Masino, Daniela Erriquez, Francesca Anna Carpagnano, Manuela Montatore, Giacomo Fascia, Alessio Sciacqua, Giuseppe Guglielmi

**Affiliations:** a Department of Clinical and Experimental Medicine, Foggia University School of Medicine, Viale L. Pinto 1, 71121, Foggia (FG), Italy; b Breast Unit, “Dimiccoli” Hospital, Viale Ippocrate 15, 70051, Barletta (BT), Italy; c Radiology Unit, “Dimiccoli” Hospital, Viale Ippocrate 15, 70051, Barletta (BT), Italy; d Radiology Unit, “IRCCS Casa Sollievo della Sofferenza” Hospital, Viale Cappuccini 1, 71013 San Giovanni Rotondo (FG), Italy

**Keywords:** Breast sarcoma, Thymoma, Mammography, Ultrasound, MRI, Case report

## Abstract

This case report aims to describe the clinical presentation, imaging findings, histopathological features and therapeutic approach of a patient diagnosed with coexisting breast sarcoma and thymoma. A 64-year-old woman presented with a palpable lump in her left breast, and subsequent imaging studies (ultrasound, mammography, and MRI) revealed breast sarcoma, a rare and aggressive subtype of breast cancer. At the same time, the MRI revealed the presence of a thymoma. A multidisciplinary approach involving surgeon, breast specialist and oncologist is essential for optimal management and favorable outcomes in patients with this rare diagnosis.

## Introduction

Breast sarcoma is a rare and aggressive subtype of breast cancer characterized by unique histological features and clinical behavior [1,2]. This malignancy represents a challenge in diagnosis and management due to its rarity and propensity for rapid progression. Unlike more common types of breast cancer, breast sarcoma is resistant to targeted therapies commonly used in breast cancer treatment. Additionally, its sarcomatoid components present diagnostic difficulties, as they resemble non-epithelial tissues and may require immunohistochemical and molecular studies for accurate classification [1,2].

Despite its rarity, breast sarcoma has garnered increasing attention in recent years due to its distinct clinical and pathological features, as well as its challenging management. This case report aims to provide a comprehensive overview of the current understanding of breast sarcoma, including its clinical presentation, imaging findings, histological characteristics, treatment strategies, and prognosis.

## Case presentation

A 64-year-old woman presented with a palpable lump in her left breast, which was noticed during self-examination. She reported no associated symptoms. She had no significant family history of breast cancer. She went through menopause at the age of 50. She reported no major surgical interventions or debilitating underlying diseases. Instead, she reported leading a sedentary lifestyle and taking a mild beta-blocker for occasional episodes of hypertension.

Further evaluation with diagnostic imaging and biopsy was warranted to determine the nature of the mass. The patient was referred for consultation with a breast specialist for further management and treatment planning. Mammography (MX) and Ultrasound (US) confirmed the presence of a suspicious area corresponding to the palpable lump.

**Fig. 1:**
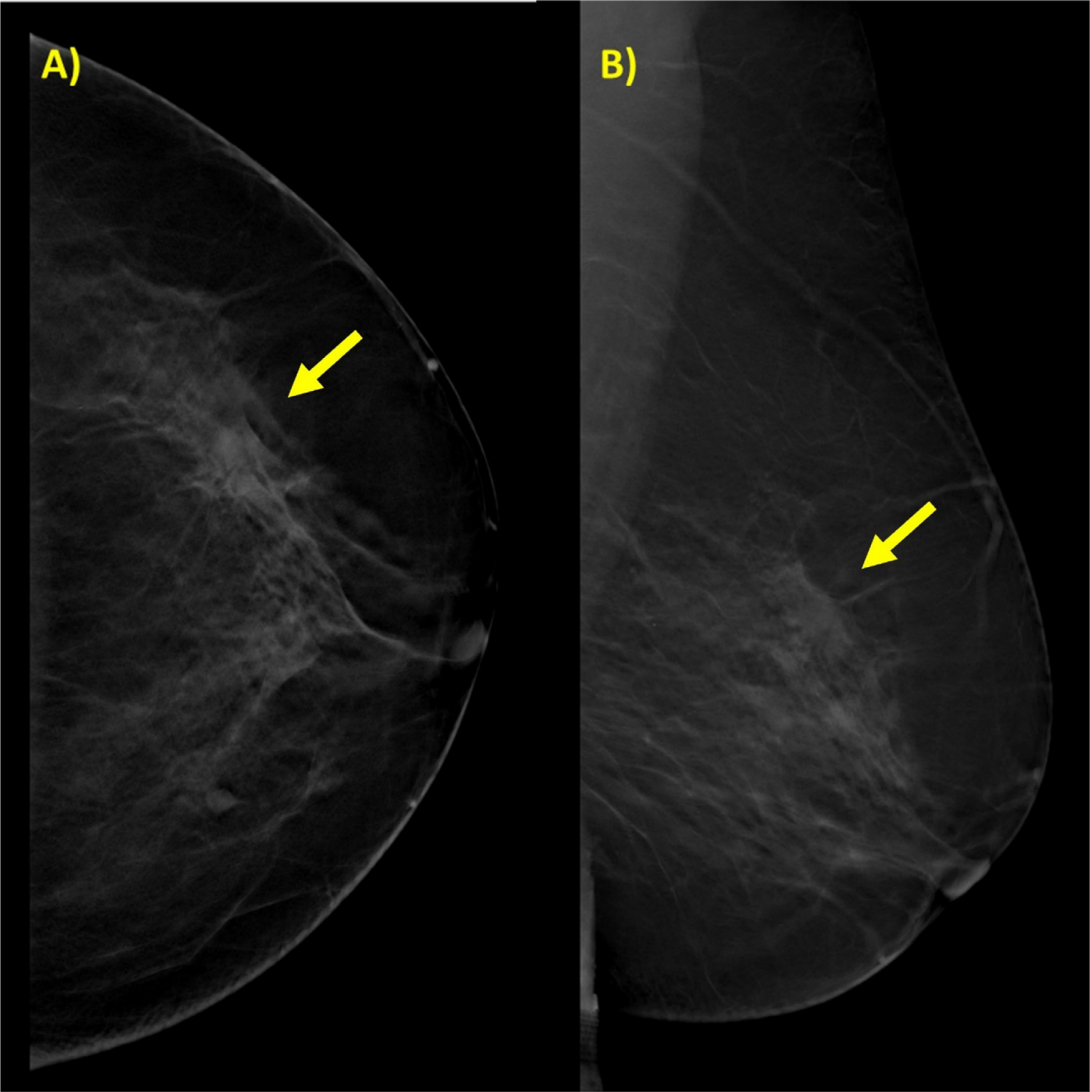
Mammography images, craniocaudal (A) and mediolateral-oblique projections (B) in the upper outer quadrant on the left breast, a radiopaque lesion associated with a parenchymal distortion (yellow arrow).

Firstly, MX revealed scattered areas of fibroglandular density within the breasts. In this context, a small irregular high-density mass with indistinct margins was observed. Few fine pleomorphic grouped intralesional calcifications were visible. Additionally, a focal architectural distortion in that area was present (Fig. 1).

**Fig. 2:**
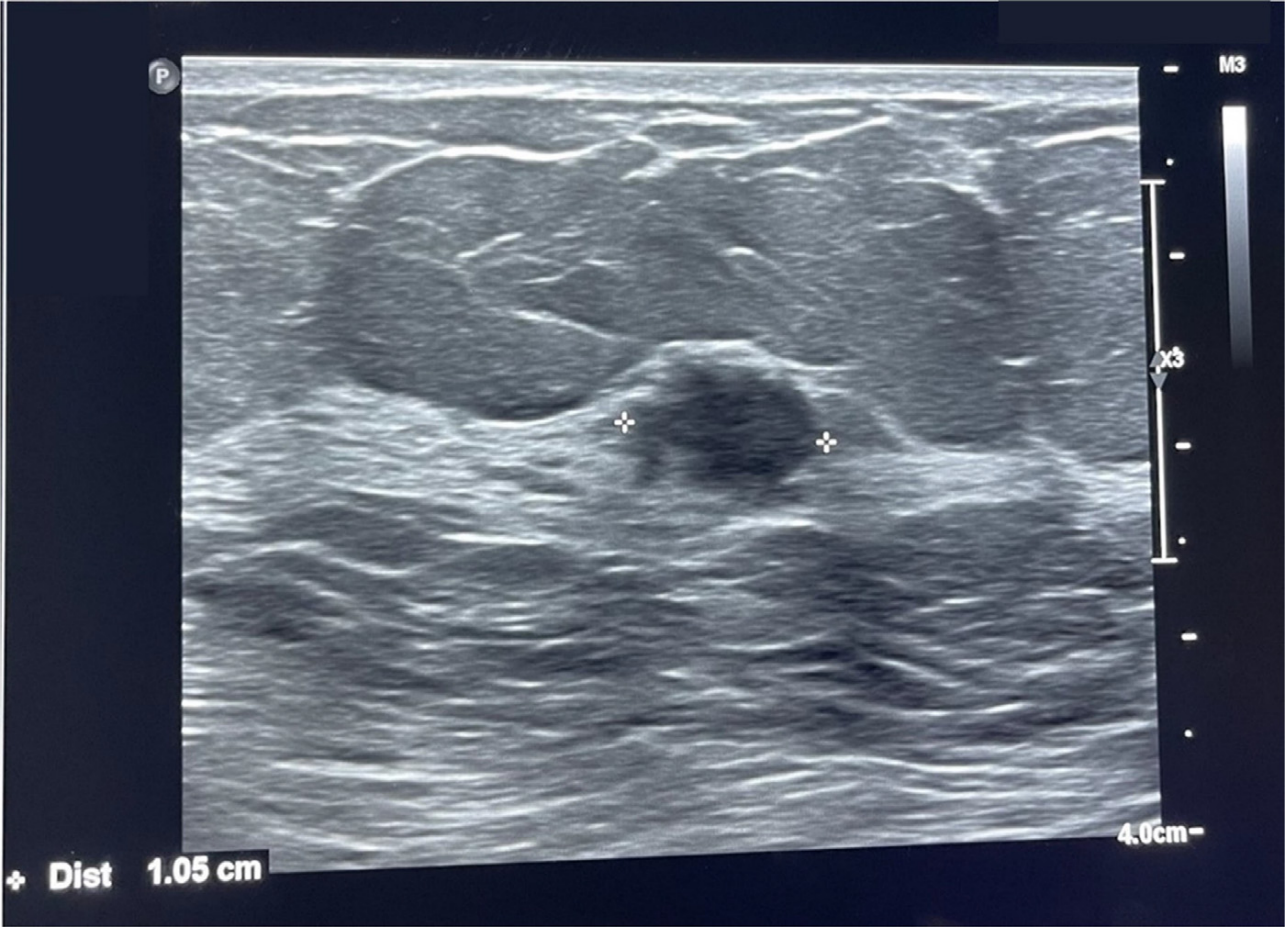
Ultrasound showed an oval-shaped formation with heterogeneous hypoechogenicity.

Subsequently, an US examination was conducted. It revealed a fibro-glandular, homogeneous background echotexture, with an oval-shaped lesion inside, appearing parallel and heterogeneous hypoechoic compared to the surrounding tissue. The mass appeared to be not well circumscribed with indistinct margins and lacked posterior features. No calcifications were visible. The lesion measured approximately 1 cm and showed a pattern of anomalous vascularity during the Color-Doppler examination. The US examination didn't detect involvement of axillary lymph nodes by the tumor. These lymph nodes appeared normal, with a thin cortex and preserved hilum, with a maximum diameter of 10 mm, not suspicious for locoregional metastatic disease (Fig. 2).

Finally, an ultrasound-guided biopsy was performed. After approximately 10 days, the histological result was of “Core needle biopsy fragments from breast glands showing high-grade malignant neoplasia with a high growth fraction (Ki67 = 70%) non-lobulocentric, with epithelioid cells and anisocariotic and pleomorphic nuclei, with a receptor profile (estrogen receptors: absent, progesterone receptors: absent, HER2: score 0) consistent with malignant mesenchymal histogenesis, namely breast sarcoma”. Histological grading was G3, indicating high proliferation of tumor cells and marked nuclear atypia. The immunohistochemical profile was as follows: positive for vimentin, Cytokeratin AE1/AE3, p63, CK5/6, CD10, weakly positive for HHF35, and E-caderina.

During subsequent blood tests, conducted by the patient independently, tumor markers revealed normal range values (Table 1).

**Table 1:** During subsequent blood tests, conducted by the patient independently, tumor markers revealed normal range values.

Tumor marker	Patient value	Normal value
CEA	1.58 ng/mL	<5 ng/mL
CA 15-3	17.1 U/mL	<30 U/mL
CA125	4.3 U/mL	<35 U/mL
CA19-9	12.2 U/mL	<37 U/mL

A dynamic contrast enhanced Magnetic resonance imaging (MRI) was performed to formally stage the tumor, and thus to exclude the presence of other foci and define the regional extent of the lesion.

**Fig. 3:**
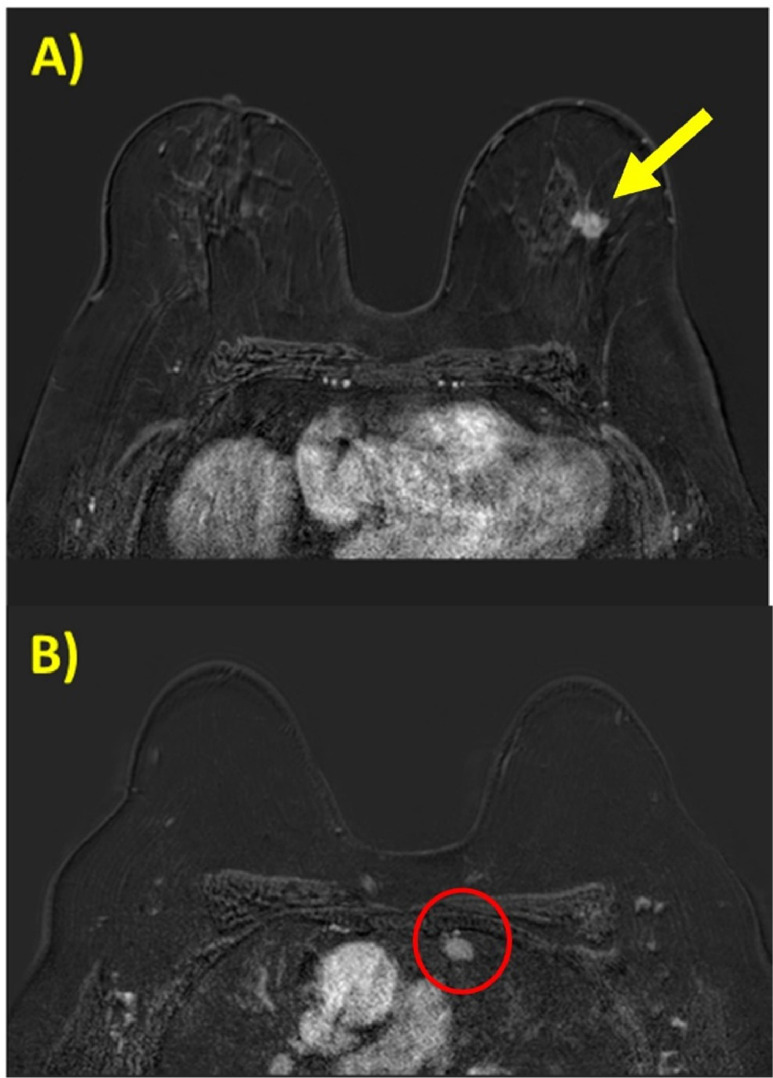
(A and B) Axial T1-weighted 3D acquisition of the first post-contrast dynamic frame showed: (A) an irregular lesion with heterogeneous mass enhancement in the upper outer quadrant of the left breast (yellow arrow); (B) a round-shaped formation with homogeneous contrast enhancement in the left side of the upper-anterior mediastinal area (red circle).

The MRI confirmed the presence of an irregular mass-like formation in the upper outer quadrant of the left breast in the context of scattered fibro-glandular tissue with mild and symmetric background parenchymal enhancement (BPE). The lesion exhibited irregular margins and measured approximately 12 mm in maximum size. It demonstrated rapid and intense contrast enhancement following the administration of Gadovist, with type III enhancement kinetics (fast initial phase and fast washout). The lesion was located 3 cm from the skin, 5 cm from the nipple-areola complex, and 5 cm from the pectoral muscle fascia. There were no pathological contrast enhancements evident in the other quadrants of the left breast and the entire right breast. Some normal lymph nodes, approximately 1 cm in size, were identified in the bilateral axillary regions, exhibiting recognizable adipose hilum and likely reactive features. Additionally, an oval-shaped formation with contrast enhancement measuring approximately 14 mm was noted as an additional finding in the upper left mediastinal area (Fig. 3).

The patient also underwent a total body CT scan with contrast medium administration to exclude the presence of metastasis. The CT examination revealed no densitometric alterations in the parenchymatous organs of the abdomen, nor in the thoracic area the presence of pathological nodules based on morpho-volumetric criteria. No secondary lesions were detected in the brain either. The bone setting did not reveal any pathological osteo-densifying or osteo-rarefying areas. On the other hand, it was confirmed the presence of a small solid nodular formation with enhancement in the adipose tissue of the left anterior mediastinum, of uncertain significance.

The neoplasm found in the adipose tissue of the anterior-superior mediastinum was removed through a left mini-thoracotomy at the fifth intercostal space. The definitive histology confirmed the presence of a thymic neoplasm consisting predominantly (90%) of a neoplastic component of type A, with a low proliferation index (Ki67 <10%), mostly composed of neoplastic epithelial cells with spindle shapes and lymphocytes. Marginally to this, there is a component with high proliferation (Ki67 = 90%), mainly consisting of immature lymphoid elements (CD3+, CD5+, TdT+, CD99+, CD1a+), and neoplastic epithelial cells with dispersed chromatin and inconspicuous nucleoli (Ck19+). The neoplasm infiltrates the adipose tissue and is focally present at the surgical resection margin (R1). The histological and immunophenotypic features are consistent with an AB-type thymoma according to WHO classification. Staging: pT1B; stage IIb1.

Subsequently, the patient underwent sentinel lymph node removal and a modifying radical mastectomy, thus sparing the pectoral muscle, and not a quadrantectomy, to avoid further radiation treatment of the thoracic area. The definitive histological examination of the surgical specimen confirmed the diagnosis of breast sarcoma (1.5 × 1.3 cm) with spindle and globoid cells in a desmoplastic, basophilic, and alcianophilic myxoid stroma in the upper outer quadrant of the left breast. Histological grading was G3, indicating high proliferation of tumor cells and marked nuclear atypia. There was no peritumoral angiolymphatic invasion. The skin, apical margins, and axillary margins of surgical resection were free from neoplasia.

Considering the dual neoplasia with evidence of R1 at the thymic level, adjuvant radiotherapy treatment to the thymic region became necessary. Following mastectomy, the patient was referred for adjuvant treatment with chemotherapy based on cytotoxic agents. Her condition was regularly monitored with imaging exams and follow-up visits. At the 2-year follow-up, there were no signs of local or metastatic recurrence.

## Discussion

The patient in this case report presented with a suspected palpable mass in the left breast. Following diagnostic workup, including imaging studies and histopathological examination of the biopsy specimen, the diagnosis of breast sarcoma was established.

This tumor type poses significant challenges in diagnosis and management due to its complex histopathological features and limited treatment options [[3], [4], [5]].

Differential diagnosis involves careful histopathological examination to differentiate between the most common breast cancers and breast sarcoma. The most common type of breast cancer is invasive ductal carcinoma (IDC), also known as infiltrating ductal carcinoma. IDC accounts for approximately 70%-80% of all breast cancer diagnoses. It originates in the milk ducts of the breast and then invades the surrounding breast tissue. Breast sarcoma, on the other hand, arises from the soft tissues of the breast, such as connective tissue, muscle, or vascular tissue. As sarcoma, IDC can also present as a lump or mass in the breast, although it can also cause changes in breast size, shape, or appearance [6,7]. While IDC is the most common type of breast cancer, there are other subtypes as well, including invasive lobular carcinoma (ILC), which accounts for about 10-15% of cases, and less common types such as inflammatory breast cancer [8,9].

Breast sarcomas includes a variety of subtypes, each with distinct histological characteristics and clinical behaviors.

First of all, from an aetiological point of view, breast sarcoma can be classified into primary and secondary. Primary occurs de novo in the breast parenchyma, secondary is usually subsequent to radiotherapy treatment with a latency period of about 5-10 years after radiotherapy. The most common type includes angiosarcoma, which arises from blood vessels. It can occur both as primary and secondary forms. Definitive diagnosis is made by biopsy with immunohistochemical analysis of specific cytokeratins [10]. In the reported case, as confirmed by histological examination and in accordance with her clinical history (absence of radiotherapy treatment), the diagnosis was of a primary type of breast sarcoma.

Metastases of primary breast sarcoma typically occur hematogenously, involving lungs, bone marrow and liver [10]. The patient fortunately showed no distant metastases. Moreover, lymphatic spread and axillary lymph node involvement are not typical of breast sarcoma, as in the case reported.

However, an enhancing nodulation in the left anterior mediastinum was identified on MRI. It was challenging to raise a diagnostic hypothesis based on imaging on the mediastinal lesion. In particular, the presence of the breast lesion could raise the hypothesis of a metastasis, but at the same time other factors such as the mediastinal location, the non-involvement of the lymph nodes, as well as the absence of metastases in the cranio-thoracic-abdominal area at CT, decreased that possibility. It was diagnosed histologically as thymoma.

The coincidence of these 2 types of tumors is not usual. At our knowledge, other cases of synchronous thymoma and breast tumors have been reported, but not particularly of sarcoma [11,12]. From a genetic point of view, the 2 tumors do not appear to be related by any particular genetic mutations [13,14]. However, while most breast sarcomas are sporadic, individuals with neurofibromatosis type 1 (NF1) or Li-Fraumeni syndrome have an increased risk of developing breast sarcomas. On the other hand, thymoma has a predisposition to the development of further neoplasms, as can occur with breast cancer [11].

The reported case is certainly useful in highlighting the importance of incidental findings in breast examination, particularly in breast MRI. In fact, meticulously examining MRI images, expanding the field of view beyond the breast, can help identify suspicious areas that deserve further evaluation, leading to early diagnosis and thus early intervention as well as customized treatment, as in the reported case.

Treatment of this aggressive tumor requires a multidisciplinary approach, with the primary goal of achieving complete resection while minimizing the risk of recurrence [[15], [16], [17]]. Due to the unexpected discovery of an additional tumor in the patient's body, namely a thymoma, radical mastectomy was performed, avoiding quadrantectomy, which would have inevitably been followed by another thoracic radiotherapy. Additionally, only radiotherapy was performed in the mediastinal area, where the previous thymoma was removed. The patient necessarily underwent chemotherapy as well, given the rare and aggressive nature of the breast tumor.

Despite aggressive management, the prognosis for patients with breast sarcoma remains guarded, with higher rates of recurrence and metastasis compared to other breast cancer subtypes. Factors such as tumor size, lymph node involvement, and response to treatment can influence long-term outcomes [[18], [19], [20]].

## Conclusion

Breast sarcoma is a rare and aggressive variant of breast cancer with distinct histopathological features and clinical behavior. A case of breast sarcoma and synchronous thymoma has rarely been described in the literature. This case highlights the importance of timely diagnosis, comprehensive staging and multidisciplinary management to optimize patient outcomes. Increasing the awareness of healthcare professionals can improve our understanding of rare cases of synchronous cancers, with the aim of improving the management of those affected.

## Authors Contribution

All authors have contributed and approved the final version of the manuscript.

## Patient consent

Complete written informed consent was obtained from the patient for the publication of this study and accompanying images.
